# p53 Rather Than β-Catenin Mediated the Combined Hypoglycemic Effect of *Cinnamomum cassia* (*L.*) and *Zingiber officinale* Roscoe in the Streptozotocin-Induced Diabetic Model

**DOI:** 10.3389/fphar.2021.664248

**Published:** 2021-05-13

**Authors:** Nasra Ayuob, Mona Ramadan Al-Shathly, Abdulaziz Bakhshwin, Nouf Saeed Al-Abbas, Nehad A Shaer, Soad Al Jaouni, Walaa H. E. Hamed

**Affiliations:** ^1^ Medical Histology and Cell Biology Department, Faculty of Medicine, Damietta University, Damietta, Egypt; ^2^ Biology Department, Faculty of Science, Jeddah University, Jeddah, Saudi Arabia; ^3^ Medical Intern, Faculty of Medicine, Alfaisal University, Riyadh, Saudi Arabia; ^4^ Biology Department, Jumum College University, Umm Alqura University, Makkah, Saudi Arabia; ^5^ Department of Chemistry, Al Leith- College, Umm Alqura University, Makkah, Saudi Arabia; ^6^ Department of Hematology/Pediatric Oncology, Yousef Abdul Latif Jameel Scientific Chair of Prophetic Medicine Application, Faculty of Medicine, King Abdulaziz University, Jeddah, Saudi Arabia; ^7^ Medical Histology and Cell Biology Department, Faculty of Medicine, MansouraUniversity, Mansoura, Egypt

**Keywords:** ginger, cinnamon, diabetes mellitus, pancreas, β-catenin, p53, antioxidants, insulin

## Abstract

**Background:** The antioxidant, hypoglycemic, and insulin-enhancing effects of ginger and cinnamon were previously confirmed in experimental and human studies, while the combined effect of ginger and cinnamon was not thoroughly investigated until now.

**Objectives:** This study was designed to assess the antidiabetic effect of combined administration of ginger (*Zingiber officinale* Roscoe) and cinnamon (*Cinnamomum cassia* L.) in streptozotocin (STZ)-induced diabetic rats compared to metformin and to explain the mechanism behind this effect.

**Materials and methods:** STZ was utilized to induce diabetes mellitus in male Sprague–Dawley rats. Assessments of fasting blood glucose level (BGL), the total antioxidant capacity (TAC), serum insulin, HOMA-IR, and HOMA–β cells were performed. Pancreatic gene expression of β-catenin and p53 was assessed using RT-PCR. Assessment of histopathological alterations of pancreatic islet cells was performed using routine and immunohistochemical techniques.

**Results:** BGL significantly decreased (*p* = 0.01), while serum insulin and TAC significantly increased (*p* < 0.001) in both metformin- and ginger plus cinnamon–treated groups compared to the untreated diabetic group. HOMA–β cell index significantly increased (*p* = 0.001) in ginger plus cinnamon, indicating their enhancing effect on insulin secretion in diabetic conditions. p53 gene expression was significantly upregulated (*p* < 0.001), while β-catenin was insignificantly downregulated (*p* = 0.32) in ginger plus cinnamon–treated groups. Insulin immunoexpression in β cells significantly increased (*p* = 0.001, *p* = 0.004) in metformin- and ginger plus cinnamon–treated groups, respectively.

**Conclusions:** The combined administration of ginger and cinnamon has a significant hypoglycemic and antioxidant effect in STZ-induced diabetes mostly through enhancing repair of islet cells mediated *via* upregulation of pancreatic p53 expression. Therefore, testing this effect in diabetic patients is recommended.

## Introduction

Diabetes mellitus (DM) is a chronic endocrine disorder that represents one of the major causes of death in humans worldwide. DM is associated with hyperglycemia which is responsible for many diabetic complications such as neuropathy, retinopathy, and nephropathy (Chaudhury et al., 2017). Hyperglycemia induces damage of DNA, lipids, and proteins, and the degree of this damage is related to the degree of oxidative stress associated with hyperglycemia (Zheng et al., 2018). Oxidative stress, induced by hyperglycemia, increases the levels of pro-inflammatory proteins and inflammatory cytokines secreted by macrophages with subsequent local and systemic inflammation (Giacco and Brownlee, 2010).

Streptozotocin (STZ) (60 mg/kg) can begin an autoimmune process that results in pancreatic β-cell toxicity with subsequent clinical diabetes within 2–4 days (Weiss, 1982). Because of that, it is used for induction of type I DM in an animal model. The latter is considered a suitable model for the evaluation of the efficiency of potential therapeutics aimed at enhancing β-cell growth and/or survival (Figeac et al., 2010). It is evidenced that β-cell mass and insulin secretion are affected in both major types of DM; hence, pancreatic β cells are targeted in new therapeutics (Nurdiana et al., 2017). Therefore, restoration of pancreatic insulin-producing β cells is vital for treating diabetes and preventing its complications.

β-catenin plays an important role in controlling glucose homeostasis. Alterations in β-catenin/Wnt signaling might increase diabetes susceptibility not only by altering the rate of β-cell proliferation and total mass but also by regulating other tissues involved in control of appetite, energy expenditure, and growth (Elghazi et al., 2012).

p53, an important tumor suppressor gene, is known as "the guardian of the genome" as it has a role in preserving stability through prevention of genome mutation. A major role of p53 in metabolism was recently highlighted (Labuschagne et al., 2018). Growing evidence showed that p53 is implicated in the pathogenesis of metabolic diseases like DM. p53 is an important regulator of glucose transport, glycolysis, and gluconeogenesis, as well as insulin resistance (Kung and Murphy, 2016). Madan et al. reported that p53 exerts its tumor suppressive activity, at least in part, through its ability to regulate glucose homeostasis (Madan et al., 2011). Stresses, like oxidative stress, result in DNA damage, activation of p53, and cell cycle arrest in order to repair or remove damaged cells (Han et al., 2008).

Many treatments of DM, including insulin secretagogues, are currently available, but most of them are associated with many unfavorable adverse effects like hypertension, diarrhea, and hypoglycemia (Inzucchi et al., 2016). Alternative natural therapeutics is the focus of recent research nowadays as they control hyperglycemia in diabetics with no or few side effects. Antioxidants were reported to improve glycemic index, guard against free radical-induced oxidative stress, and decrease the incidence of many complications (Aisa et al., 2019).

Ginger (Zi*ngiber officinale* Roscoe), a worldwide popular herb belongs to the family Zingiberaceae, is used as a spice. The popularity of ginger goes back 3,000 years and it was appreciated by Arab merchants for whom the value of ginger was important (El-Seedi et al., 2019). Many studies were conducted to clarify ginger's influence on glucose and lipid metabolism. The hypoglycemic, antioxidant, and androgenic effects of ginger was recently confirmed (Al-Shathly et al., 2020).

Cinnamon (*Cinnamomum cassia*), a flavoring agent belongs to the family Lauraceae, is commonly used in traditional medicine as it has noteworthy insulin-potentiating, antioxidant, and anti-inflammatory properties (Qin et al., 2014). It was reported that cinnamon improved anthropometric parameters, lipid profile, and glycemic indices of type II diabetic patients (Zare et al., 2019). Although the hypoglycemic effect of ginger (Akash et al., 2015; Carvalho et al., 2020) and cinnamon (Sahib, 2016; Santos and da Silva, 2018) administrated separately were frequently studied in both animal and human, the hypoglycemic effect of combined ginger and cinnamon administration was not sufficiently studied. Therefore, this study was designed to evaluate the effect of the combined administration of ginger and cinnamon on the glycemic, antioxidant capacity, and the structure of pancreatic islet cells in an animal model of diabetes mellitus compared to metformin and to explore the mechanism behind this effect. We hypothesized that when administrated together, ginger and cinnamon could induce significant anti-hyperglycemic and antioxidant effects in STZ-induced DM. This combined effect might be mediated through upregulation of pancreatic p53 and β-catenin expression.

## Materials and Methods

### Drugs

Streptozotocin, purchased from Sigma Aldrich Chemical Company (CO. St. Louis, MO, United States), was utilized for induction of DM in rats (60 mg/kg). It was dissolved in 0.01 M sodium citrate buffer, pH 4.5 before the immediate intraperitoneal injection (Salemi et al., 2016).

Metformin hydrochloride was purchased from Shanghai Medicine Co., Ltd., China, and was utilized for pharmacological validation of ginger and cinnamon. It was dissolved in 0.9% (W/V) sodium chloride at a dose of 500 mg/kg/day and administrated through gastric gavage for 6 weeks (Pushparaj et al., 2000).

Fresh roots of ginger (*Zingiber officinale* Roscoe) (voucher specimen: AQJ_84) and *Cinnamomum cassia*, L (voucher specimen: AQJ_15) were purchased from the local market at Jeddah, Saudi Arabia. They were identified in the King Abdulaziz University (KAU) herbarium using specimens of herbarium, Flora of Kingdom of Saudi Arabia (Chaudhary et al., 1999). Voucher specimens were deposited in the herbarium and the identification of the plants was verified by a botanist from Faculty of Science, KAU.

The watery extract of ginger was prepared as previously described by Al-Amin et al. (2006) and stored in a refrigerator at 4°C. It was dissolved in normal saline and orally administrated through a gastric gavage at a dose of 500 mg/kg body weight (bw)/day for six weeks. This dose was selected as it was reported to induce a significant impact when orally administrated (Thomson et al., 2002; Al-Amin et al., 2006). LD50 of *Zingiber officinale* was estimated to be 4,525.5 mg/kg in rats (Abdulrazaq et al., 2012).

The aqueous extract of cinnamon was prepared according to the method previously described (Longe et al., 2015). It was dissolved in normal saline and orally administrated through the gastric gavage at the dose of 100 mg/kg of bw/day for 6 weeks as this dose was reported to induce a significant hypoglycemic effect of cinnamon at this dose (Longe et al., 2015). Cinnamon extract below 0.5 g/kg dose level is safer to be used in the efficacy study (Ahmad et al., 2015).

### GC–MS Analysis of the *Zingiber officinale* Roscoe and *Cinnamomum cassia* L Extracts

The chemical composition of both plants were analyzed using Trace Gas Chromatography GC-TSQ evo 8,000 mass spectrometer (Thermo Scientific, Austin, TX, United States) with a direct capillary column TG–5MS (30 m × 0.25 mm x 0.25 µm film thickness). The column oven temperature was initially held at 50°C and then increased by 5°C/min to 250°C, held for 2 min, and increased to the final temperature to 300°C by 25°C/min, and held for 2 min. The injector and MS transfer line temperatures were kept at 270, 260°C, respectively. Helium was used as a carrier gas at a constant flow rate of 1 ml/min. The solvent delay was 4 min and diluted samples of 3 µl were injected automatically using Autosampler AS1300 coupled with GC in the splitless mode in a PTV injector. EI mass spectra were collected at 70 eV ionization voltages over the range of m/z 50–650 in a full-scan mode. The ion source temperature was set at 250°C. The components were identified by comparison of their mass spectra with those of WILEY 09 and NIST 14 mass spectral database that is used in identification and study the chemical composition of unknown components in any extract (Wiley, 2006). Analysis had been done in qualitative type using Thermo Scientific™ Xcalibur™ 2.2 software, and all values were reported in relative percentage (El-Kareem et al., 2016).

### Animals

We confirmed that all methods were carried out in accordance with relevant guidelines and regulations. This study was reviewed and approved by the biomedical research ethics committee at the Faculty of Medicine, KAU, Jeddah, KSA. A total of thirty male Sprague–Dawley rats weighing 100–150 g and aged 5 weeks ± 3 days were purchased from the animal unit at King Fahed Medical Research Center, KAU, Jeddah, KSA. Rats were housed in plastic cages in an air-conditioned room under the standard laboratory conditions at 22 ± 1°C. They were offered the standard animal chow and water ad libitum and left to be acclimatized for one week. The rats were assigned into equal 5 groups (*n* = 6). The normal control group (NC) received normal saline through a gastric tube and the treated control group (ginger + cinnamon) received a mixture of ginger and cinnamon extracts orally at 1:1 ratio. The other three groups were administrated freshly prepared STZ after being fasted for 12 h followed by 5% sucrose solution overnight to avoid hypoglycemia and facilitate STZ entry to β cells (Islam and Choi, 2008). After 7 days, blood was obtained from the tail vein of all rats by using Bayer's Contour meter (Japan), and the blood glucose level (BGL) was measured. Rats with BGL > 250 mg/dl were considered diabetic (Nie et al., 2002). The diabetic rats were further assigned to 3 groups (*n* = 6) including the untreated diabetic (STZ), metformin-treated (STZ + metformin), and ginger plus cinnamon-treated (STZ + ginger + cinnamon) groups. All treatments were continued for six weeks. Rats were individually weighed at the beginning and at the end of the experiment by using a sensitive balance and their weights were recorded in grams.

### Biochemical Assessment

At the end of the experiment, the rats were fasted for 12 h, then blood samples were obtained from the retro-orbital vein and centrifuged at 3,000 rpm for 15 min for serum separation that was stored at −80°C. The enzymatic glucose kits were used to assess the BGL (Human Gesellschaft für and Diagnostica mbH, Germany). Insulin ELISA kits were used to measure serum insulin levels (Cat. no. ezrmi-13kelisa, Billerica, MA, United States) according to the previously described method (Gurudeeban et al., 2016).

The homeostatic model assessment for insulin resistance (HOMA-IR) and homeostatic model assessment for β cells (HOMA–β cells) were calculated as was previously described (Matthews et al., 1985) based on these formulas: HOMA-IR = fasting serum glucose (mg/dl) × fasting serum insulin (μU/ml)/405.

HOMA–β-cell function = 20 x fasting serum insulin (μU/ml)/fasting serum glucose (mg/dl)−3.5.

The total antioxidant capacity was estimated using the colorimetric method according to the guidelines of the kit used (Bio-diagnostic, Giza, Egypt) (Koracevic et al., 2001).

### Gene Expression Assessed Using Real-Time RT-PCR

Rats were anesthetized with 4% isoflurane (SEDICO Pharmaceuticals Company, Cairo, Egypt) in 100% oxygen and then euthanized by cervical dislocation. The thorax was opened and the cardiac perfusion was performed with 10% neutral buffered formalin in order to allow for the rapid fixation of the organs and prevent the occurrences of the degenerative changes. The abdomen was opened and pancreatic tissues were quickly dissected out, fixed in 10% neutral buffered formalin for 24 h, and processed to obtain paraffin blocks. Parts of paraffin-processed samples were subjected to RNA extraction according to the method previously described (Pikor et al., 2011). Extraction of total RNA using TRIzol was done based on the supplier instruction (Invitrogen Life Technologies, Carlsbad, CA, United States). Nano Drop 2000 Spectrophotometer (Thermo Scientific, United States) was used to measure the concentration of RNA. Reverse transcription was done using oligo-dT primers (Bioneer Inc. Daejeon, Republic of Korea) in a 20-μl reaction including 5 μl RNA. The resulted cDNAs were amplified using PCR Master Mix (Bioneer Inc. Daejeon, Republic of Korea) with primers (Metabion International AG, Semmelweisstr, Germany) (Pikor et al., 2011). β-catenin, forward 5′-TGC​TGA​AGG​TGC​TGT​CTG​TC-3′ and reverse 5′-TCG GTA ATG TCC CTG TC-3′; p53, forward 5′-ATT​TCA​CCC​TTA​AGA​TCC​GTG​GG-3′ and reverse 5′-AGA​CTG​GCC​CTT​CTT​GGT​CT-3′; β-actin, forward 5′-CCC ATC TAT GAG GGT TAC GC and reverse 5′-TTT AAT GTC ACG CAC GAT TTC-3′ were used. The results analysis was done using the LightCycler 480 software (version 1.5, Roche Applied Science, Mannheim, Germany). The relative levels of mRNA were analyzed using the ΔΔCt method.

### Histopathological Assessment

The paraffin blocks of the pancreas were sectioned at 4 μm and processed to be stained with hematoxylin and eosin for histopathological examination using a light microscope (Olympus Microscope BX-51, German) connected to a digital camera.

Another set of paraffin sections were immunohistochemically stained using an avidin–biotin technique that was described by Goto et al. (2015). After being incubated with 10% normal goat serum, paraffin sections were incubated with anti-p53 antibody (rabbit monoclonal antibody Santa Cruz, United States, at dilution 1:400), anti–β-catenin (rabbit polyclonal antibody against β-catenin; Santa Cruz, United States, at dilution 1:400), and anti-insulin (mouse monoclonal antibody, Dako, Denmark, at a dilution of 1:100) at 4°C overnight. The sections were further processed for developing the color with diaminobenzidine tetrahydrochloride supplemented with 0.04% hydrogen peroxidase and counterstained with Mayer’s hematoxylin.

In the pancreas, the areas of 10 islets were measured in three serial H&E–stained sections at a magnification of ×100. The mean area percent of insulin and β-catenin immunoexpression as well as the number of p53 positive cells were assessed in at least 20 islets per rat at a magnification ×100 using Pro Plus image analysis software version 6.0 as was previously described (Afifi, 2012).

### Statistical Analysis

Data were analyzed using Statistical Package for the Social Sciences (SPSS) version 16. One-way analysis of variance followed by least significant difference (LSD) post hoc test was used to compare the variables of the studied groups. Body weight and blood glucose level were affected by two independent variable factors: effect of time (start and end of study, paired) and effect of treatment (unpaired), and therefore were analyzed using a mixed-model 2-way ANOVA. Data were presented as mean ± SD. *p* value less than 0.05 was considered to be significant.

## Results

### Analysis of the Ginger and Cinnamon Extracts

The constituents of *Zingiber officinale* Roscoe *and Cinnamomum cassia (L.)* were determined using gas chromatography and mass spectrometer (GC–MS). It was noticed that *Zingiber officinale* Roscoe mainly includes zingiberene (∼10%), citral (∼9%), cedrene (∼5%), β-sesquiphellandrene (∼5%), cinnamaldehyde (∼4%), and other components (Table 1). It was noticed that cinnamon mainly includes cinnamaldehyde (∼87%), coumarin (∼10%), and other components. It was noticed that many compounds of the two extracts have hypoglycemic, antidiabetic, anti-inflammatory, and antioxidant activities (Table 1).

**TABLE 1:** Components of *Z. officinale* Roscoe and *Cinnamon* extract identified using gas chromatography and mass spectrometer (GC–MS) analysis.

Compounds	Retention time	Relative %	Activity
*Z. officinale* Roscoe			
Zingiberene	13.41	10.16	Antioxidant activity El-Ghorab et al. (2010)
			Anti-inflammatory and antiapoptotic effect Li et al. (2020)
Citral	19.16	8.48	Antidiabetic activity Mishra et al. (2019)
			Anti-inflammatory effect Martins et al. (2017)
			Antihyperlipidemic effect Najafian et al. (2011)
Cedrene	26.21	5.51	Anti-inflammatory effect Jeong et al. (2014)
β-Sesquiphellandrene	15.13	5.23	Antioxidant activity Zhao et al. (2010)
			Antiproliferative activity Tyagi et al. (2015)
Cinnamaldehyde	30.11	4.16	Hypoglycemic and hypolipidemic effects Subash Babu et al. (2007)
			Antidiabetic effect Zhang et al. (2008)
Cederanol	19.74	3.83	No activity reported
α-Farnesene	14.51	2.19	Anticancer activity Afoulous et al. (2013)
Juniper camphor	30.41	1.89	No activity reported
Curcumene	15.70	1.70	Antitumor activity Itokawa et al. (1985)
Bergamotene	23.75	1.49	No activity reported
β-Bisabolene	13.16	1.37	Antiproliferative activity Jou et al. (2016)
Nuciferol	33.52	0.99	No activity reported
Carotol	33.39	0.89	Antifungal activity Jasicka-Misiak et al. (2004)
α-Terpineol	16.01	0.74	Antioxidant and anti-inflammatory activities Sousa et al. (2020)
Giranol	19.26	0.67	No activity reported

*Cinnamomum cassia*			
Cinnamaldehyde	30.40	86.73	Hypoglycemic and hypolipidemic effects Subash Babu et al. (2007)
			Antidiabetic effect Zhang et al. (2008)
Coumarin	42.02	9.40	Antidiabetic effect Li et al. (2017)
			Anti-inflammatory and antioxidant effect Hadjipavlou-Litina et al. (2007)
o-Methoxycinnamaldehyde	40.93	1.97	Anti-inflammatory effect Hadjipavlou-Litina et al. (2007)
			Antiapoptotic activity Golshahi et al. (2019)
Benzenepropanol	28.28	0.59	No reported activity
Ar-turmerone	33.06	0.74	Antidiabetic activity Kuroda et al. (2005)
			Anti-inflammatory effect Park et al. (2012)
Benzalmalonic dialdehyde	25.13	0.35	No activity reported

### Effect of Ginger and Cinnamon Extracts on Mean Body Weight

**FIGURE 1:**
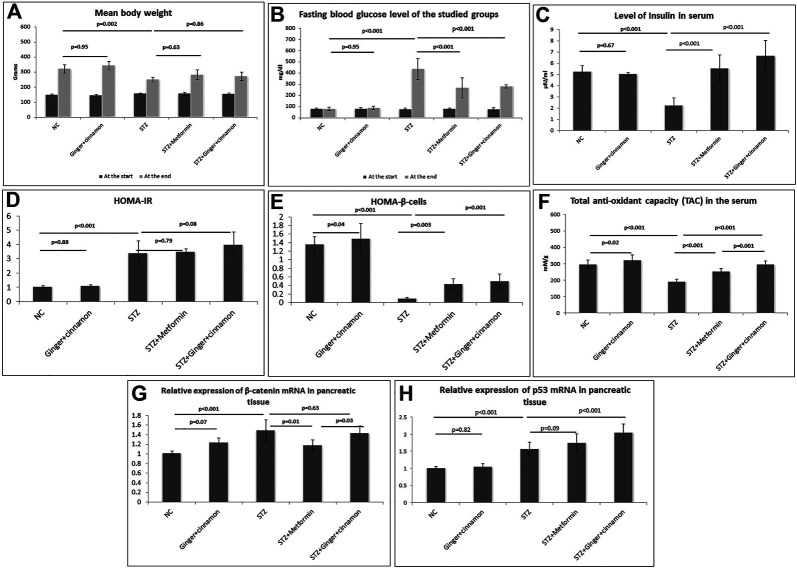
Body weight **(A)**, fasting blood glucose level **(B)**, serum insulin **(C)**, HOMA-IR **(D)**, HOMA–β cells **(E)**, total antioxidants capacity **(F)**, relative expression of β-catenin mRNA **(G),** and p53 mRNA **(H)** in the pancreatic tissue of the studied groups are presented. NC: normal control and STZ: streptozotocin. Data are presented as the mean ± SD, *n* = 6. Comparison between groups was done using one-way ANOVA test followed by LSD post hoc test.

There was no significant difference between the mean body weights of the rats in all the studied groups at the start of the experiment. Although there was no significant difference between the rats that received ginger plus cinnamon and the control rats at the end of the experiment, STZ-treated rats showed significantly lower (*p* = 0.002) body weight compared to the control. No significant difference in body weight was observed in metformin-treated and ginger plus cinnamon–treated groups when compared to the STZ group Figure 1A.

### Effect of Ginger and Cinnamon Extracts on the BGL and Serum Insulin Levels

To assess the hypoglycemic effect of the combined administration of ginger and cinnamon, estimation of fasting BGL was performed. At the beginning of the experiment, assessing the fasting BGL revealed normoglycemic levels in all studied groups. On the 7th day of STZ injection, the BGL was elevated to more than 250 mg/dl in all rats. The BGL significantly increased (*p* < 0.001) in the STZ group compared to the normal control group, while it significantly decreased in metformin- (*p* < 0.001) and ginger plus cinnamon–treated (*p* < 0.001) groups compared to the STZ group with no significant difference between the two groups (Figure 1B).

A significant reduction (*p* < 0.001) in serum insulin was recorded in the STZ group, while treatment with metformin as well as ginger plus cinnamon significantly increased (*p* < 0.001) insulin levels, with a significant difference (*p* = 0.04) between the two treated groups (Figure 1C).

### Effect of Ginger and Cinnamon Extracts on HOMA-IR and HOMA–β Cell

The HOMA-IR and HOMA–β cells were calculated in order to assess insulin resistance and β-cell function, respectively. Although HOMA-IR was significantly increased in the STZ group compared to the normal control, it showed no significant change in either metformin- (*p* = 0.79) nor ginger plus cinnamon–treated groups compared to the STZ group (Figure 1D). When it came to HOMA–β cells, there was a significant increase (*p* = 0.04) in control rats that received ginger plus cinnamon, while it was significantly reduced (*p* < 0.001) in the STZ group compared to the normal control. Treatment with either metformin (*p* = 0.003) or ginger plus cinnamon (*p* = 0.001) significantly increased HOMA–β cell with a significant difference (*p* = 0.001) between the two treated groups (Figure 1E).

### Effect of Ginger and Cinnamon Extracts on Total Antioxidant Capacity

In order to assess the effect of the combined administration of ginger and cinnamon on the antioxidants status, TAC was measured. It was observed that the level of TAC in the rats that received ginger plus cinnamon was significantly higher (*p* = 0.02), while that of the STZ group was significantly lower (*p* < 0.001) than the normal control rats. On the other hand, treatment of diabetic rats with metformin or ginger plus cinnamon significantly (*p* < 0.001) increased the TAC level compared to the STZ group with a significant (*p* = 0.001) difference between the two groups (Figure 1F).

### Effect of Ginger and Cinnamon Extracts on β-Catenin and p53 Gene Expression in Pancreatic Tissue

Gene expression of β-catenin was assessed in pancreatic tissue using RT-PCR in order to explore their role in glucose homeostasis. It was found that the mRNA level of β-catenin was significantly upregulated (*p* < 0.001) in the STZ group relative to the normal control. Although mRNA level of β-catenin was significantly downregulated (*p* < 0.001) in the metformin-treated group, it was insignificantly downregulated (*p* = 0.32) in the ginger plus cinnamon–treated group compared to the STZ group (Figure 1G).

When it came to gene expression of p53 in the pancreatic tissue, it was observed that its level was significantly upregulated (*p* < 0.001) in the STZ group relative to the normal control, and it was further upregulated in both metformin- (*p* = 0.09) and ginger plus cinnamon–treated groups (*p* < 0.001) compared to the STZ group with a significant difference (*p* = 0.01) between the two treated groups. See (Figure 1H).

### Effect of Ginger and Cinnamon Extracts on the Histological Structure of the Pancreas

**FIGURE 2:**
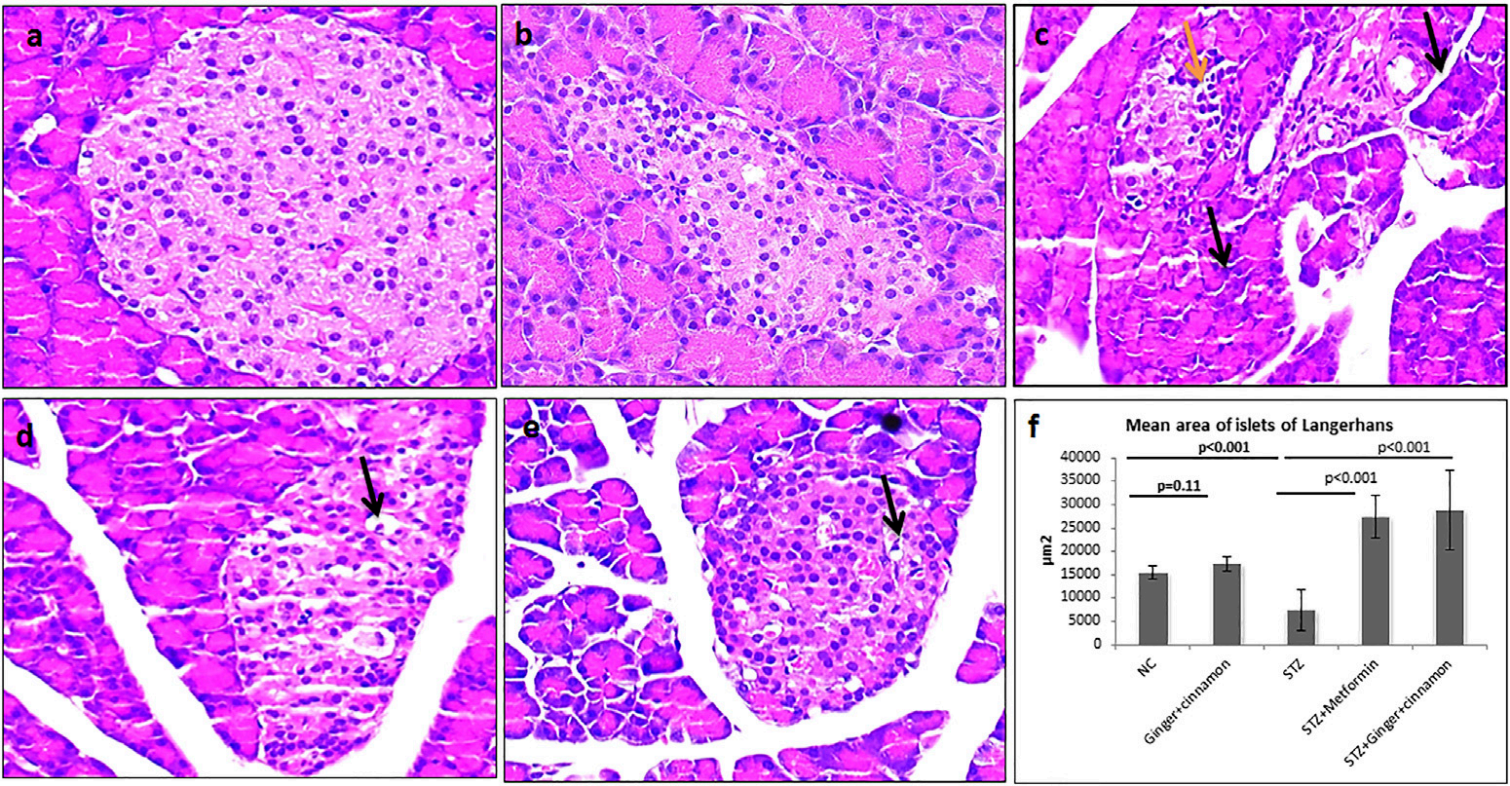
Photomicrographs of H&E–stained pancreas of normal control **(A)** and ginger plus cinnamon–treated control **(B)** showing normal architecture of the pancreas included the exocrine component and the lightly stained islets of Langerhans. Pancreas of diabetic untreated rat **(C)** showing some shrunken acini with small dark nuclei and cytoplasm (black arrow). Many islet cells are vacuolated (yellow arrow). Pancreas of diabetic rat treated with metformin **(D)** or ginger plus cinnamon **(E)** showing preserved general architecture with most of the acinar and islet cells appear more or less intact apart from few vacuolated cells (H&E × 400). **(F)**: Graph shows the mean area of islets of Langerhans of the studied groups. The area of islets was measured in 10 non-overlapping islets (H&E) per rat. Data are presented as the mean ± SD, *n* = 6. Comparison between groups was done using one-way ANOVA test followed by LSD post hoc test. Pro Plus image analysis software version 6.0 was used. https://www.mediacy.com/imageproplus.

Histopathological and immunohistochemical techniques were performed to assess the effect of the combined administration of ginger and cinnamon on the structure of islet cells. The pancreatic islets of Langerhans of the normal control and ginger plus cinnamon–treated groups appeared intact, while those of the diabetic group showed some pathological changes as many of the islet cells appeared vacuolated with dark nuclei. On the other hand, pancreas of metformin- or ginger plus cinnamon–treated diabetic rats showed that most of the islet cells appeared intact apart from few vacuolated cells, which were less frequently observed in the ginger plus cinnamon–treated group. Statistical analysis showed a significant decrease (*p* < 0.001) in the mean area of islets of Langerhans in the STZ group compared to the normal control while that of the metformin- or ginger plus cinnamon–treated group were significantly increased (*p* < 0.001) compared to the STZ group with no significant difference (*p* = 0.45) between the two treated groups (Figure 2).

### Effect of Ginger and Cinnamon Extracts on Immunoexpression of β-Catenin, p53, and Insulin

**FIGURE 3:**
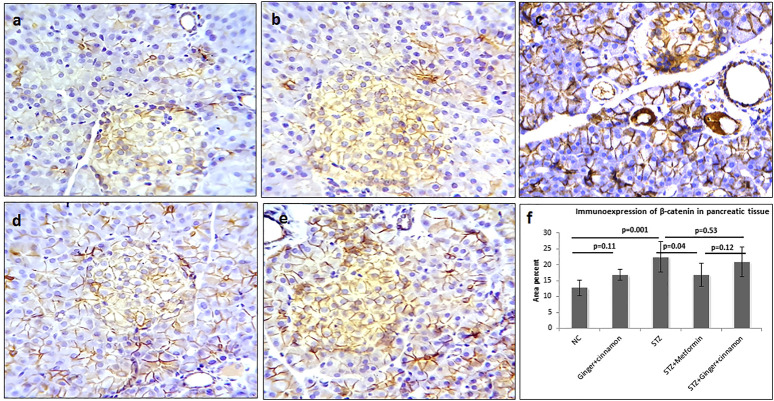
Photomicrographs of pancreas stained immunohistochemically with anti–β-catenin antibody showing mild expression in normal control **(A)**, ginger plus cinnamon **(B)**, diabetic rat treated with metformin **(D),** and diabetic rat treated with ginger plus cinnamon **(E)**, while that of that diabetic untreated rat **(C)** showing strong β-catenin expression (anti–β-catenin antibody × 400). **(F)**: graph showing the area percent of β-catenin immunoexpression in the studied groups. NC: normal control and STZ: streptozotocin. Data are presented as the mean ± SD, *n* = 6. The area percent is measured in at least 20 islets per rat. Compression between groups was done using one-way ANOVA test followed by LSD post hoc test. Pro Plus image analysis software version 6.0 was used. https://www.mediacy.com/imageproplus.

Pancreas of control and ginger plus cinnamon–treated control groups showed mild β-catenin immunoexpression in the lateral borders of acinar, ductal, and islet cells, while the STZ group showed a significant increase (*p* < 0.001) in β-catenin expression in comparison to the normal control. Treatment with metformin significantly reduced (*p* = 0.004) β-catenin immunoexpression, while treatment with ginger plus cinnamon induced an insignificant decrease (*p* = 0.13) compared to the STZ group (Figure 3).

**FIGURE 4:**
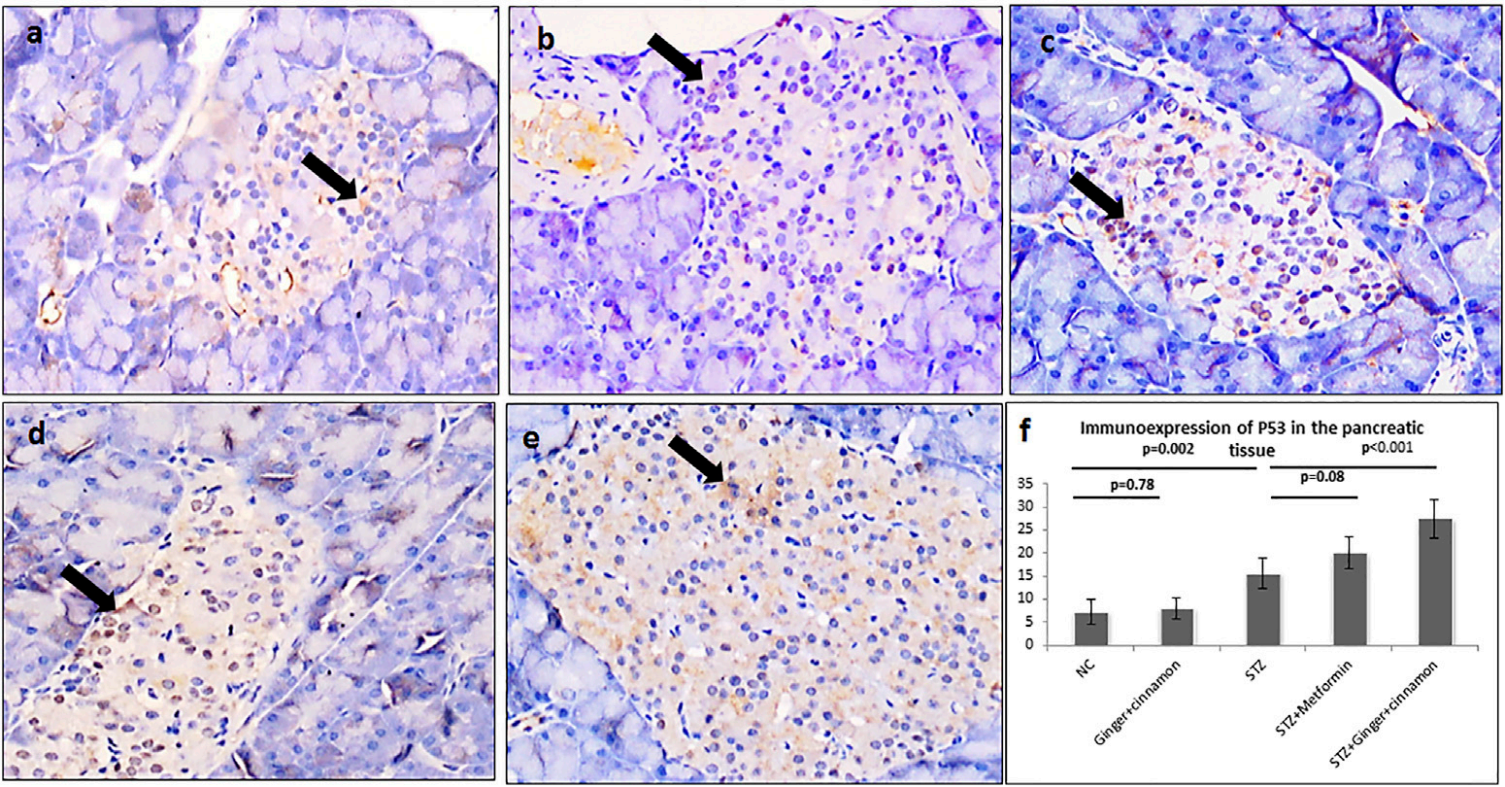
Photomicrographs of pancreas stained immunohistochemically with anti-p53 antibody showing few number of p53 +ve cells in the normal control group **(A)**, ginger plus cinnamon–treated group **(B)**, diabetic group treated with metformin **(D)**, and diabetic group treated with ginger plus cinnamon **(E)**, while that of that diabetic untreated rat **(C)** showing increased number of these cells (anti-p53 antibody × 400). **(F)**: Graph showing the mean number of p53 +ve cells in the studied groups. NC: normal control and STZ: streptozotocin. Data are presented as the mean ± SD, *n* = 6. The area percent is measured in at least 20 islets per rat. Comparison between groups was done using one-way ANOVA test followed by LSD post hoc test. Pro Plus image analysis software version 6.0 was used. https://www.mediacy.com/imageproplus.

Regarding immunoexpression of pancreatic p53, there were few p53-positive cells in the pancreatic islets of the control and ginger plus cinnamon–treated groups, while those of the STZ group showed significantly higher (*p* < 0.001) number of p53-positive cells than the control. Treatment with metformin and ginger plus cinnamon resulted in an increase (*p* = 0.08, *p* < 0.001) in the number of p53-positive cells, respectively, compared to the STZ group (Figure 4).

**FIGURE 5:**
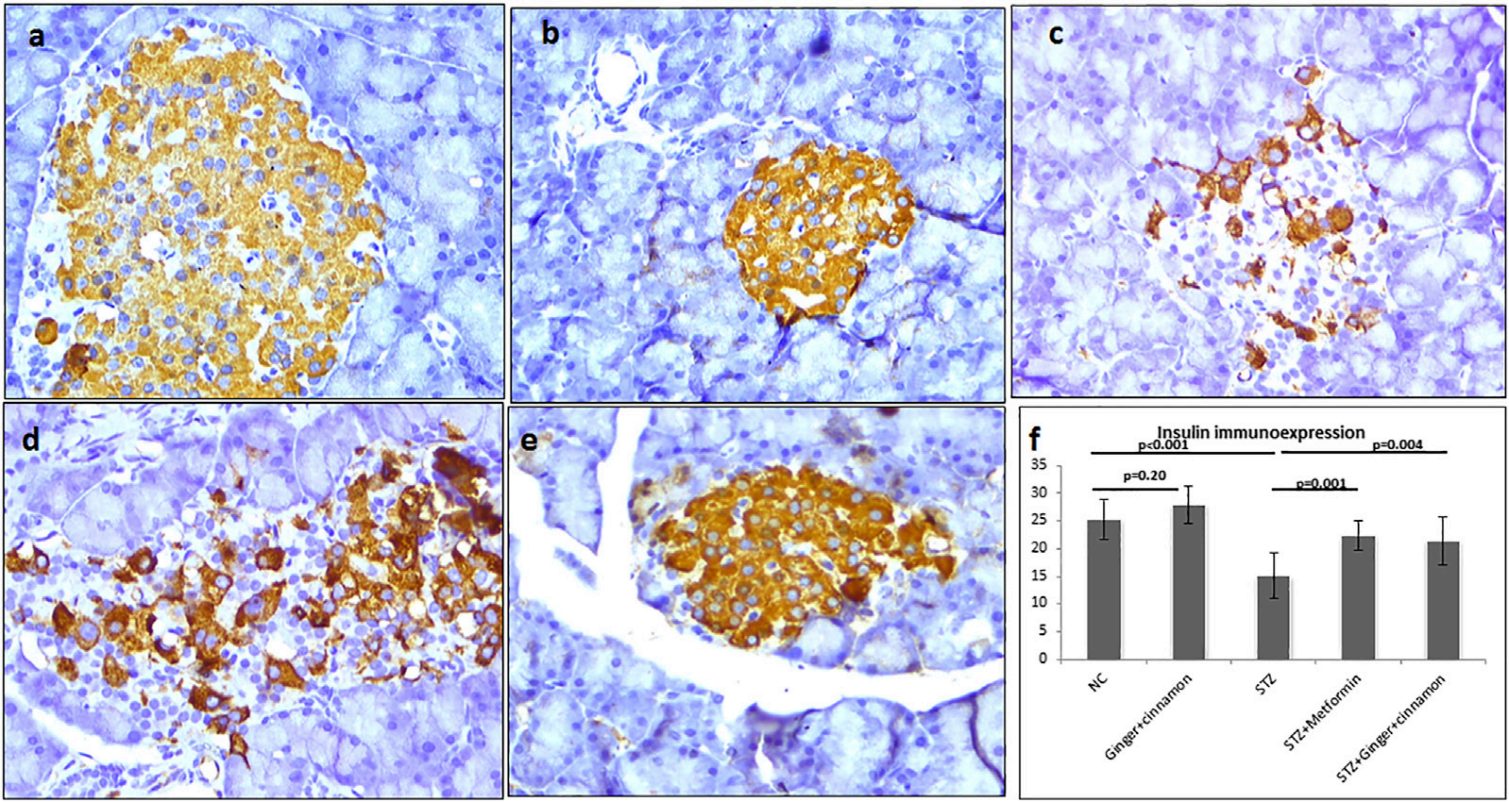
Photomicrographs of pancreas stained immunohistochemically with anti-insulin antibody showing moderate expression in almost all β cells of the normal control **(A)**, ginger plus cinnamon **(B)**, and diabetic rat treated with ginger plus cinnamon **(E)**, while that of that diabetic untreated rat **(C)** showing moderate expression in few β cells and diabetic rat treated with metformin **(D)** showing moderate expression in many β cells (anti-insulin antibody × 400). **(F)**: graph showing the area percent of insulin immunoexpression in the studied groups. NC: normal control and STZ: streptozotocin. Data are presented as the mean ± SD, *n* = 6. The area percent is measured in at least 20 islets per rat. Comparison between groups was done using one-way ANOVA test followed by LSD post hoc test. Pro Plus image analysis software version 6.0 was used. https://www.mediacy.com/imageproplus.

Strong immunoexpression of insulin was observed in β cells of both control and ginger plus cinnamon–treated control groups. Statistical analysis revealed a significant reduction (*p* < 0.001) in insulin immunoexpression in β cells of the STZ group compared to the control, while those of metformin- or ginger plus cinnamon–treated groups showed a significant increase (*p* = 0.001, *p* = 0.004) compared to the STZ group, respectively (Figure 5).

## Discussion

Diabetes mellitus is a common chronic health problem which is highlighted for the severity of its complications (Cortez et al., 2015). Hyperglycemia is considered the predominant cause behind diabetic complications. Despite optimal treatment regimens, fluctuations in blood glucose level in diabetics remain a major challenge (Monnier and Colette, 2015). Diabetes-associated oxidative stress that was postulated as a possible mechanism for diabetes-associated tissue damage and systemic complications represents another challenge in managing DM (Madsbad, 2016). Therefore, it was concluded that replenishment of insulin-producing pancreatic β cells is crucial for treating diabetes and preventing its complications (Nurdiana et al., 2017). In this study, the antidiabetic effect of combined ginger and cinnamon was investigated in the STZ-induced animal model of diabetes and the mechanism behind this effect was explored. In addition, the impact of these two plants on insulin-producing pancreatic β cells was investigated.

In this study, administration of ginger plus cinnamon to diabetic rats reduced the progression of hyperglycemia comparable to that of metformin. This anti-hyperglycemic effect was evident by the significant decrease in fasting BGL compared to the untreated diabetic group and was associated with a significant increase in serum insulin and HOMA–β-cell index indicating their enhancing effect on insulin section in diabetic conditions. On the other hand, HOMA-IR index showed no significant change after treatment with ginger plus cinnamon indicating no effect on insulin sensitivity. The significant increase in insulin immunoexpression as well as preservation of islet cells structure was considered confirming findings of augmented insulin secretion in the diabetic rats treated with ginger and cinnamon. These findings were supported by the previous researches that studied the anti-hyperglycemic effect of ginger and cinnamon separately. In this study, analysis of ginger revealed many compounds with hypoglycemic (citral and cinnamaldehyde), anti-inflammatory (zingiberene, citral, cedrene, and α-terpineol), and antioxidant (zingiberene, β-sesquiphellandrene, and α-terpineol) effects. Analysis of cinnamon extract also revealed many compounds with hypoglycemic (cinnamaldehyde, coumarin, and ar-turmerone), anti-inflammatory (coumarin, o-methoxycinnamaldehyde, and ar-turmerone), and antioxidant (coumarin) effects, which confirmed and explained its antidiabetic effect observed in this study.

Ginger was reported to reduce glucose levels through its ability to increase glucose uptake and glycogen synthesis as well as increasing the phosphorylation of the insulin receptor. Ginger also promoted glucose clearance in insulin-responsive peripheral tissues and augmented insulin release which maintained blood glucose homeostasis. Additionally, ginger can prevent STZ-induced oxidative stress by inhibiting lipid peroxidation and hence protect β cells from the damaging effect of diabetes-induced free radicals (Alshathly 2019). Steamed ginger extract was recently proven to have anti-hyperglycemic efficacy in alloxan-induced type 1 diabetic mice by closing KATP channels in pancreatic β cells and subsequent stimulation of insulin secretion (Nam et al., 2020). Similarly, the efficacy of ginger was reported in type 2 diabetic patients as demonstrated by reduced serum insulin level and enhanced insulin sensitivity compared to placebo (Arablou et al., 2014; Zhu et al., 2018). Regarding cinnamon, it was reported to significantly reduce the blood glucose levels by increasing the levels of insulin and adiponectin through expression of peroxisome proliferation-activated receptors (PPARγ) in adipose tissue (Kim and Choung, 2010). In a more recent study, cinnamon extract was reported to have insulin-like effect that causes inhibition of hepatic glucose production and decreased gene expression of enzymes involved in hepatic gluconeogenesis (Al-Waili et al., 2017). This could explain increased serum insulin observed in the current study, following the combined administration of both ginger and cinnamon.

Uncontrolled DM, in animal and human, is characterized by the occurrence of oxidative stress. The persistent high blood glucose level in conditions of uncontrolled DM results in glycation of the antioxidant enzymes with subsequent reduction in their activity (Al-Attar and Alsalmi, 2019). Many factors are included in the pathophysiological changes implicated in the development of diabetic complications in human and animal. Among these factors is enhanced generation of reactive oxygen species (ROS) due to oxidative stress (Robson et al., 2018). Therefore, the combined anti-hyperglycemic effect of two plants with potent antioxidant activity was studied in this work. In this study, it was found that the combined administration of ginger and cinnamon significantly increase the TAC in the diabetic rats. The improvement observed in diabetes patients and experimental animals after treatment with ginger was attributed to the potent scavenging effect of free radicals induced by ginger active constituents like gingerol, vallinoids, paradol, and zingerone (El-Ghawet et al., 2019). Adding to that, cinnamon is rich in procyanidins and catechins that have potent antioxidant activities which diminish oxidative stress and guard against beta cells damage, and this might be behind the antidiabetic effect of cinnamon (Al-Waili et al., 2017).

In this study, both immuno- and gene expression of β-catenin was upregulated in STZ-induced diabetic rats, and significantly downregulated in metformin-treated rats. These findings were in accordance with those of Murad et al. (2015). They found that metformin decreased the inappropriate β-catenin expression induced by high-fat diet/low-dose STZ, while they attributed the metformin-induced regeneration of islets to the better control of diabetes and decrease of oxidative stress (Murad et al., 2015). There exists an interaction betweenglucagon-like peptide 1 (GLP-1) and Wnt/β-catenin signaling pathway. Activation of signaling pathway of GLP-1 receptor is important for repair of deficient β-cell mass, and Wnt signaling appears to mediate GLP-1–induced β-cell proliferation (Chiang et al., 2012). Both *in vivo* and *in vitro* studies of diabetic rat models as well as complementary techniques revealed the implication of Wnt/β-catenin signaling in the neonatal growth and regeneration of pancreatic β cells as well as mature β-cell function and survival (Figeac et al., 2010).

It was reported that 6-gingerol of *Zingiber officinale* rhizome upregulated the protein expression and mRNA levels of β-catenin detected by Western blotting and real-time PCR, respectively (Li and Zhou, 2015). On the other hand, cinnamaldehyde, the primary chemical constituent of the cinnamon, was reported to downregulate β-catenin expression detected by real-time PCR and Western blotting (Wu et al., 2017). In other words, ginger and cinnamon have an opposing effect on β-catenin expression. Therefore, the combined administration of ginger and cinnamon resulted in an insignificant upregulation of β-catenin in the control rats and an insignificant downregulation of β-catenin in diabetic rats. It seems that the preserved integrity of the islet’s cells and their increased insulin content as well as increased antioxidant capacity observed in this study following the combined administration of ginger and cinnamon is behind the better control of diabetes and not through the Wnt/β-catenin pathway.

In this study, STZ induced a significant upregulation of pancreatic p53 immuno- and gene expression compared to the control. This finding was in agreement with the study of Al-Jarrah et al. who found that the level of p53 expression in the heart muscle of diabetic rats increased significantly compared to the control rats (Al-Jarrah et al., 2012). Tornovsky-Babeay et al. reported that glucotoxicity results in DNA damage as well as p53 activation, which finally induces beta cell death and aggravates diabetes (Tornovsky-Babeay et al., 2014). Kung and Murphy added that p53-mediated senescence of adipocytes and pancreatic beta cells is associated with the development of insulin resistance and diabetes (Kung and Murphy, 2016). On the other hand, increased p53 expression might be directly attributed to STZ-induced hyperglycemia or indirectly to STZ-associated oxidative stress (Adeyemi and Adewole, 2019). In addition, ROS can upregulate p53 to induce p53-mediated apoptosis of pancreatic beta cells (Yuan et al., 2010). It was mentioned that among the causes that lead to enhanced p53 expression is increased oxidative stress (Dashtiyan et al., 2017). In mouse models of both type 1 and type 2 diabetes, increased endoplasmic reticulum (ER) stress stimulates nuclear exportation and cytosolic accumulation of p53 that ends finally with mitochondrial dysfunction and insulin deficiency (Hoshino et al., 2014).

The effect of ginger on p53 expression was previously detected in some studies. Steam-distilled extract of ginger was investigated for its efficacy in treating endometrial cancer as it mediates apoptosis by activating p53 (Liu et al., 2012). Pashaei-Asl et al. reported that treatment with ginger resulted in upregulation of p53 protein, downregulation of BCL-2, and inhibition of ovarian tumor cells (Pashaei-Asl et al., 2017). In addition, cinnamon was previously described to enhance the expression of p53. It was reported that pretreatment with eugenol, a phenolic compound that is naturally present in cinnamon, significantly increased p53 expression that augmented apoptosis in the 7,12-dimethylbenz [a] anthracene (DMBA)–induced neoplastic skin lesion (Kaur et al., 2010). These findings explain the significant increase in p53 expression recorded, in this study, after the combined treatment with ginger plus cinnamon, while metformin induced an insignificant upregulation of p53 expression. Upregulation of p53 results in stopping of cell cycle in order to allow for cell repair and survival or removal of damaged cells (Han et al., 2008).

In conclusion, this study suggested that when administrated together, ginger and cinnamon induced significant anti-hyperglycemic and antioxidant effects and could preserve the endocrine pancreas against STZ-induced toxicity. Combined ginger and cinnamon administration might exert these effects through upregulation of pancreatic p53 expression that helps pancreatic β-cell survival or removal of damaged cells adding to the better control of diabetes and antioxidant effect. β-catenin did not show a significant role in explaining this effect. Therefore, the combination of ginger and cinnamon is recommended to be tested for efficacy on diabetic patients either as an antidiabetic or adjuvant therapy.

## Data Availability

The raw data presented in the study is included in the article/Supplementary Material, further inquiries can be directed to the corresponding author.

## Abbreviations

DM, diabetes mellitus; MDM2, mouse double minute 2; MHCP, methyl-hydroxychalcone polymer; STZ, streptozotocin; BGL, blood glucose level; HOMA-IR, homeostatic model assessment for insulin resistance; PPARγ, peroxisome proliferation-activated receptors; ROS, reactive oxygen species; GLP-1, glucagon-like peptide 1; PCR, polymerase chain reaction; ER, endoplasmic reticulum; DMBA, the 7,12-dimethylbenz [a] anthracene.
